# Long term clinical and MRI follow-up of 76 patients after synovial cyst resection via microscopic hemilaminectomy: a retrospective study

**DOI:** 10.1016/j.bas.2025.105630

**Published:** 2025-10-09

**Authors:** Jan Lodin, Jan Terč, Filip Cihlář, Alena Sejkorová, Martin Sameš, Petr Vachata

**Affiliations:** a Neurosurgical Department, J. E. Purkynje University, Masaryk Hospital Krajská Zdravotní a.s., Sociální Péče 3316/12A, Ústí nad Labem, Czech Republic; b Faculty of Medicine in Pilsen, Charles University in Prague, Husova 3, Plzeň, Czech Republic; c Department of Radiology, J. E. Purkynje University, Masaryk Hospital Krajská Zdravotní a.s., Sociální Péče 3316/12A, Ústí nad Labem, Czech Republic

**Keywords:** Synovial cyst, Instability, Resection, Hemilaminectomy, Spondylolisthesis

## Abstract

**Introduction:**

Synovial cysts can be treated by several non-instrumented surgical techniques, which are commonly grouped into non-homogenous cohorts. Few studies have analyzed surgical results of synovial cyst resection via microscopic hemilaminectomy with adequate imaging and long-term follow-up.

**Research question:**

What is the long-term rate of reoperation, synovial cyst recurrence, new instability, listhesis progression, new synovial cysts in unoperated segments and postoperative patient function after synovial cyst resection via microscopic hemilaminectomy?

**Materials and methods:**

Seventy-six patients who underwent synovial cyst resection by microscopic hemilaminectomy were analyzed via a postoperative MRI, dynamic radiographs, clinical examination and Oswestry Disability Index (ODI). Dependent variables were rate of reoperation, new instability, listhesis progression, cyst recurrence, postoperative patient function and new stenosis/synovial cysts in unoperated segments. They were plotted against independent variables including age, intervertebral disc degeneration, indirect signs of instability and surgical invasiveness.

**Results:**

Reoperation was performed in 10 cases after a mean period of 71.3 months. Cyst recurrence occurred in 4 cases, new instability in 21 cases and listhesis progression in 37 cases. New synovial cysts and stenosis occurred in 6 and 9 cases respectively and 63 patients reported a postoperative ODI ≤40. Preoperative spondylolisthesis was associated with listhesis progression, new instability, higher postoperative ODI and new stenosis.

**Discussion and conclusion:**

Treatment of synovial cysts by microscopic hemilaminectomy yields acceptable long-term results. Preoperative spondylolisthesis appears to affect postoperative ODI, instability and spondylolisthesis but not the reoperation rate or cyst recurrence. No other independent variable appeared to affect dependent variables.

## Highlights

• Reoperation rate of synovial cyst resection via hemilaminectomy is 13.2 %.
• Preoperative spondylolisthesis is associated with higher postoperative ODI.
• New asymptomatic instability or listhesis after surgery occurred in 49 % and 28 % of cases respectively.

## 1 Introduction

Juxta-articular facet cysts (or simply synovial cysts) of the lumbar spine are benign well circumscribed extradural lesions found in close proximity to the facet joints, which can be encountered incidentally or due to clinical symptoms including, back pain, radicular pain or neurological deficits (Sabo et al., 1996; Rosenstock and Vajkoczy, 2020). They are commonly regarded as a marker of spinal instability and have divided opinion in whether they necessitate instrumentation for successful treatment (Ramhmdani et al., 2019; Kulkarni et al., 2017). This resulted in a multitude of studies and metanalyses comparing instrumented and non-instrumented procedures throughout the last decade. Although several reviews have demonstrated comparable results of both surgical modalities, they are limited by several fundamental flaws (Garg and Kasliwal, 2021; Benato et al., 2023; Gonzalez et al., 2023). These include combining several non-instrumented modalities with variable destabilizing potential into one category, unclear guidelines of when a spine segment is deemed unstable and short follow-up periods without the use of objective tools such as imaging or standardized questionnaires. The following study attempted to limit these flaws by performing one type of surgical procedure on a patient population with clearly defined spine stability. Furthermore, follow-up was performed using the validated Oswestry Disability Index together with a clinical exam and postoperative dynamic X-rays and MRI to definitively rule out iatrogenic instability, synovial cyst recurrence or other complications. Minimal follow-up was set at 2 years in order to demonstrate the longevity of treatment. Primary endpoints of our study were base postoperative characteristics of the patient cohort including reoperation rate, percentage of new instability, degree of listhesis progression, number of cyst recurrences, postoperative patient function (Oswestry Disability Index) and new stenosis/synovial cysts in unoperated adjacent segments. Secondary endpoints included analysis of the dependency of base postoperative characteristics on independent variables including age, body mass, index, facet joint edema, disc height, Phirrmann disc degeneration, presence of listhesis, cyst volume and degree of facet joint drilling.

## 2 Methods

### 2.1 Study design and selection criteria

The following is a single center, retrospective observational study consisting of 76 patients who underwent synovial cyst resection without instrumentation between January 2008 and January 2021. Patients were required to have complete hospital records consisting of flexion/extension radiographs, an MRI of the lumbar spine, a baseline neurological examination and a complete medical history. Isolated cyst resection was only performed if the motion segment containing the cyst was deemed stable, otherwise the patient underwent instrumented stabilization and was excluded from the study. For purposes of this study, instability was defined as a difference in anterolisthesis between lateral flexion/extension radiographs over 3 mm or a difference in anterolisthesis between lateral standing radiographs and a lumbar spine MRI over 3 mm. Similarly, angular changes within a motion segment between flexion/extension radiographs >15° were considered unstable. Measurements were performed both by a radiologist and the operating surgeon and the segment was deemed unstable if at least one dynamic measurement exceeded our cut-off value of 3 mm or 15°. Indirect signs of instability such as facet joint edema, disc height or coronal facet angles did not play a role in determining segment stability. Patients were excluded if the surgical invasiveness exceeded solitary cyst resection. This included patients who underwent additional microdiscectomy, multi-level surgery or resection of multiple cysts. In all cases, the surgical approach was a microscopic hemilaminectomy and partial abrasion of the adjacent facet joint. In cases of purely foraminal cysts a contralateral approach was used, otherwise an ipsilateral hemilaminectomy was performed. The degree of laminar and facet drilling was determined individually by each surgeon based on the type, size and location of the synovial cyst. Patients underwent a standard 4-day postoperative physiotherapy protocol and were prescribed a further 10 rounds of physiotherapy at release. The minimal follow-up period was 2 years, after which patients underwent a control MRI, flexion/extension radiographs, and a clinical examination along with the Oswestry Disability Index (ODI) questionnaire.

### 2.2 Analyzed parameters

**Fig. 1:**
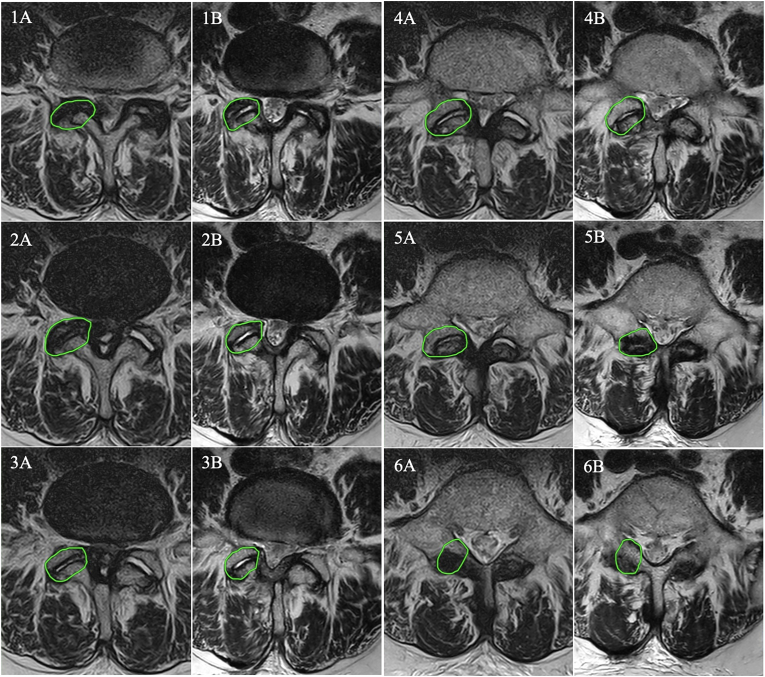
Comparison of facet joint volume on preoperative MRI imaging (left side A, 15.9 ml) and postoperative imaging (right side B, 15.3 ml).

Several radiographic variables were analyzed by a radiologist. Disc height was measured as the mean average between the highest and lowest sagittal disc height and its Pfirrmann grade was recorded from 1 to 5. Cyst volume was estimated by multiplying maximal height, width and length measurements of the cyst. The facet joint width was calculated by measuring maximal articular space width of both facets and adding both values. The degree of listhesis was determined by measuring the anterior slippage of the cranial vertebra on a standing radiograph. Parameters analyzed on postoperative imaging included progression of listhesis, measured as the difference in slippage between preoperative and postoperative standing radiographs, new instability, new listhesis, synovial cyst recurrence or newly developed stenosis/listhesis/synovial cysts in adjacent motion segments. Furthermore, the volume of facet joint drilling was quantified by comparing preoperative and postoperative MRI imaging by a board-certified radiologist and measuring volume of the facet joint in each slice using the Marie WebVision software (OR-CZ spol. s r.o.) (Fig. 1). In cases of symptomatic cyst recurrence or newly developed instability a secondary instrumented procedure was performed.

### 2.3 Statistical methods

All statistical analysis was performed by Statistika 13.0 (TINCO software Inc., Palo Alto, California) software by an external statistician. Dependent variables (DV) included the reoperation rate, percentage of new instability, degree of listhesis progression, number of cyst recurrences, postoperative patient function (Oswestry Disability Index) and new stenosis or synovial cysts in unoperated adjacent segments. Independent variables (IV) were age at time of surgery, BMI, height and Phirrmann degeneration of the intervertebral disc, cyst volume, facet edema, presence of preoperative listhesis and degree facet joint abrasion. Multivariate analysis was used to determine which IV affected DV. Significant relationships between DV with IV were calculated using either the Mann-Whitney test (age at time of surgery, BMI, disc height, cyst volume, facet edema) or Pearson's chi-squared test (Phirrmann degeneration, presence of preoperative listhesis and degree facet joint abrasion). Relationships between variables were considered statistically significant if *p* were less than 0.05.

## 3 Results

Basic demographic information and preoperative clinical characteristics are summarized in Table 1. The final cohort consisted of 76 patients (43 women, 33 men) with a mean age 67.9 years. Most patients presented with radiculopathy (96.1 %), the remaining 3 presented with a combination of back pain and neurogenic claudication. A total of 74 of cysts were located between L3-S1 segments, only 2 cysts were located between L1-3. Mean cyst volume was 1.27 ml and 12 cysts were classified as large with a volume above 2 ml. Preoperative spondylolisthesis was present in 39.8 % of cases with a mean slippage of 1.9 mm. All operated segments were deemed stable by both a radiologist and neurosurgeon with an inter-observer percentage agreement of 93.4 %, which represented a strong agreement by Cohen's kappa (κ = 0.82). Surgery was performed within 1 day–9 months of the onset of symptoms via a microscopic hemilaminectomy and partial facetectomy if necessary. A complete hemilaminectomy was necessary in 13 cases (17.1 %), most commonly due to large cysts with extensive cranial or caudal propagation. In 10 cases (13.2 %), it was necessary to drill over 50 % of the facet joint in order to gain sufficient access to the cyst, in the remaining cases over 50 % of the facet joint was preserved. No intraoperative complications such as dural defects, subtotal resection or radicular damage occurred. Table 2 demonstrates basic information at follow-up. Follow-up was performed at a mean interval of 93 (25–178) months. Revision surgery was necessary in 10 cases (13.2 %) and was performed on average 71.3 (21–178) months after primary surgery and typically within one month of symptom recurrence. Reasons for secondary instrumentation were either cyst recurrence (4 cases) or newly developed instability (6 cases). In 37 cases, follow-up standing radiographs demonstrated progression of listhesis or a new listhesis, however only 6 cases were symptomatic due to concurrent instability. Similarly, 21 patients demonstrated new instability, however 15 cases were asymptomatic without significant back pain, neurogenic claudication or radiculopathy. Interestingly, 6 patients developed new synovial cysts in an adjacent segment and 9 patients developed new spinal stenosis in an adjacent segment. None of the adjacent synovial cysts were symptomatic, however subsequent microdecompression was performed in 3 cases of adjacent segment stenosis. Residual radicular pain was present in 30 patients, however pain levels decreased from a mean NRS of 8.3 to 4.5.

**Table 1:** Demographic and clinical patient data (n = 76).

**Age of surgery (mean)**	67.9	**Facet joint drilling**	
**Male**	*33 (44.4 %)*	*< 50 %of the facet joint*	*66 (86.8 %)*
**Female**	*43 (56.6 %)*	*> 50 % of the facet joint*	*10 (13.2 %)*
**Preoperative pain (mean NRS)**	8.3	**Width of facet joint effusions**	

**Clinical symptoms**		*≤*4 mm	38 (50 %)

*Hypesthesia*	*16 (21.1 %)*	*>*4 mm	38 (50 %)
*Paresis*	*21 (27.6 %)*	**Spondylolisthesis present**	30 (39.8 %)
*Neuroclaudication*	*17 (22.4 %)*	1 mm	3 (3.9 %)

***Radicular pain***	***73 (96.1 %)***	2 mm	19 (25 %)
*L4*	*14 (18.4 %)*	3 mm	*6 (7.9 %)*
*L5*	*36 (47.4 %)*	*>*4 mm	*2 (2.6 %)*
*S1*	*23 (30.3 %)*		

**Cyst level**		**Phirmann grade**	
*L1/2*	*1 (1.3 %)*	*1*	*19 (25 %)*
*L2/3*	*1 (1.3 %)*	*2*	*44 (57.9 %)*
*L3/4*	*11 (14.5 %)*	*3*	*12 (15.8 %)*
*L4/5*	*48 (63.2 %)*	*4*	*1 (1.3 %)*
*L5/S1*	*15 (19.7 %)*		

**Cyst type**		**Cyst volume (mL)**	
*Articular*	*70 (92.1 %)*	*Small (<1)*	*39 (51.3 %)*
*Flavar*	*4 (5.3 %)*	*Medium (1–2)*	*25 (32.9 %)*
*Foraminal*	*2 (2.6 %)*	*Large (>2)*	*12 (15.8 %)*

**Table 2:** Results after surgical decompression (n = 76).

**Cyst recurrence**	4 (5.3 %)
**Listhesis progression**	37 (48.7 %)

**New instability**	21 (27.6 %)

**New cyst in adjacent segment**	6 (7.9 %)

**New stenosis in adjacent segment**	9 (11.8 %)

**Oswestry disability index**	

*0–20*	*42 (55.3 %)*

*21–40*	*21 (27.6 %)*

*41–60*	*11 (14.5 %)*
*61–80*	*2 (2.6 %)*
*81–100*	*0*

**Residual radicular pain/mean NRS**	30 (39 %)/4.5

**Secondary instrumentation**	10 (13.2 %)

**Mean time period of revision surgery (months)**	71.3

### 3.1 Statistical relationships between dependent and independent variables

Multivariate analysis of dependent and independent variables is shown in Table 3. No statistically significant relationships were found between reoperated patients and independent variables. Although 60 % of reoperated patients demonstrated preoperative stable listhesis, this did not reach statistical significance (*p* = 0.10). The only independent variable significantly affecting dependent variables was the presence of preoperative stable listhesis in the segment containing the synovial cyst. Compared to patients without preoperative listhesis, patients presenting with a listhesis were more likely to for it to progress (73 % vs 33 %), develop new instability (50 % vs 13 %), develop new spine stenosis in an adjacent segment (13 % vs 10 %) and had significantly higher postoperative Oswestry Disability Indexes (mean 27.9 vs 19.3). However, preoperative listhesis did not significantly affect cyst recurrence, the genesis of new cysts in adjacent segments and ultimately reoperation rate.

**Table 3:** Multivariate analysis of dependent and independent variables.

		Independent variables							
		*Age at surgery*	*BMI*	*Disc height*	*Phirrmann grade*	*Cyst volume*	*Facet edema*	*Preoperative listhesis*	*Facet drilling*
**Dependent variables**	*Reoperation*	0.51	0.74	0.79	0.85	0.25	0.47	0.10	0.75
	*New instability*	0.42	0.32	0.18	0.68	0.44	0.87	<0.001	0.35
	*Cyst recurrence*	0.65	0.11	0.39	0.55	0.94	0.51	0.98	0.42
	*Listhesis progression*	0.11	0.68	0.57	0.44	0.44	0.78	<0.001	0.44
	*ODI*	0.13	0.64	0.68	0.48	0.56	0.77	<0.001	0.30
	*Stenosis adjacent*	0.11	0.09	0.43	0.48	0.07	0.61	0.04	0.39
	*Cyst adjacent*	0.53	0.89	0.62	0.95	0.47	0.63	0.94	0.79

## 4 Discussion

The optimal management of symptomatic spinal facet cysts remains elusive resulting in a plethora of metaanalyses and reviews throughout the last decade (Garg and Kasliwal, 2021; Benato et al., 2023; Gonzalez et al., 2023). These reviews, although very thorough, have not yielded definitive conclusions regarding a superior surgical strategy. In our view, this is due to several shortcomings of their input studies. These include a) a clear definition of the surgical procedure performed and quantification of surgical invasiveness, b) a clear definition of when a motion segment is deemed unstable and c) short follow-ups without adequate postoperative imaging, resulting in underestimated rates of long-term complications. A review of major studies investigating non-instrumented decompression procedures was conducted by the authors and is detailed in Table 4 (Kulkarni et al., 2017; Page et al., 2021; Rolemberg Dantas et al., 2020; Wun et al., 2019; Campbell et al., 2018; Bruder et al., 2017; Knafo et al., 2015; James et al., 2012; Jankowski et al., 2012; Landi et al., 2012; El Shazly and Khattab, 2011; Xu et al., 2010; Weiner et al., 2007; Deinsberger et al., 2006; Khan et al., 2005; Metellus et al., 2006; Sandhu et al., 2004; Pirotte et al., 2003; Trummer et al., 2001; Lyons et al., 2000; Domenicucci et al., 2017; Scholz et al., 2017; Vergara et al., 2017; Birch et al., 2016; Denis et al., 2016; Chesney et al., 2022; Sukkarieh et al., 2015; Akbary et al., 2020; Hwang et al., 2017; Komp et al., 2014; Tacconi et al., 2020; Oertel and Burkhardt, 2017; Epstein, 2004). The Pubmed and Scopus databases were searched for terms ‘synovial’, ‘cyst’, ‘spine’ and ‘surgery’. Studies between years 2000 and 2024 were included if they included a minimum of 10 follow-up patients. The aim of our study was to retrospectively analyze the results of a homogenous surgical procedure performed on a clearly defined patient population with a sufficiently long follow-up including X-ray and MRI imaging, which allowed quantification of surgical invasiveness.

**Table 4:** Review of major clinical studies focusing on non-instrumented synovial cyst resection.

	Study	Patient number	Type of surgical decompression	Reoperation rate	Type of follow-up	Follow-up in months (mean)
**Variable surgical modalities**	Page (2020) (Page et al., 2021)	161	Variable - hemilaminectomy to multiple laminectomies	10.5 %	Clinical	85.2
	Rolemberg Dantas (Rolemberg Dantas et al., 2020) (2020)	50	Variable – interlaminar to hemilaminectomy	6 %	Clinical	87.6
	Wun (2019) (Wun et al., 2019)	55	Variable - hemilaminectomy to laminectomy	11.5 %	Clinical	65.1
	Campbell (2018) (Campbell et al., 2018)	158	Variable - hemilaminectomy to laminectomy	10.8 %	Clinical	Unclear
	Bruder (2017) (Bruder et al., 2017)	141	Variable – interlaminar to laminectomy	11.3 %	Clinical + ODI	111
	Knafo (2015) (Knafo et al., 2015)	21	Variable – interlaminar to laminectomy	4.8 %	Clinical	17.6
	James (2012) (James et al., 2012)	16	Variable - hemilaminectomy to laminectomy	0 %	Clinical	14
	Jankowski (2012) (Jankowski et al., 2012)	11	Variable – interlaminar to hemilaminectomy	0 %	Clinical + X-rays	12
	Landi (2011) (Landi et al., 2012)	15	Variable – interlaminar to hemilaminectomy	0 %	Clinical + X-ray	8.3
	El Shazly (2011) (El Shazly and Khattab, 2011)	13	Variable - hemilaminectomy to laminectomy	7.7 %	Clinical	50.4
	Xu (2010) (Xu et al., 2010)	90	Variable - hemilaminectomy to laminectomy	3 %	Clinical	16.5
	Weiner (2007) (Weiner et al., 2007)	23	Hemilaminectomy	15 %	Clinical	116.4
	Deinsberger (2006) (Deinsberger et al., 2006)	30	Variable - hemilaminectomy to laminectomy	0 %	Clinical	12
	Khan (2005) (Khan et al., 2005)	13	Laminectomy	38 %	Clinical	26
	Metellus (2005) (Metellus et al., 2006)	77	Variable - hemilaminectomy to laminectomy	2.6 %	Clinical	42
	Sandhu (2004) (Sandhu et al., 2004)	17	Hemilaminectomy	5.9 %	Clinical	12.8
	Pirotte (2003) (Pirotte et al., 2003)	42	Variable – interlaminar to laminectomy	9.5 %	Clinical	Unclear
	Trummer (2001) (Trummer et al., 2001)	19	Variable - hemilaminectomy to laminectomy	0 %	Clinical	22.7
	Lyons (2000) (Lyons et al., 2000)	194	Variable - hemilaminectomy to laminectomy	9.3 %	Clinical	26
**Hemilaminectomy**	Domenicucci (2017) (Domenicucci et al., 2017)	22	Hemilaminectomy open	4.5 %	Clinical + X-ray + MRI	28.5
	Scholtz (2017) (Scholz et al., 2017)	74	Hemilaminectomy (tubular/open)	12.2 %	Clinical + ODI	69
	Vergara (2017) (Vergara et al., 2017)	24	Hemilaminectomy (tubular)	0 %	Clinical	9.4
	Birch (2016) (Birch et al., 2016)	40	Hemilaminectomy (tubular)	5 %	Clinical + X-ray	79
	Denis (2016) (Denis et al., 2016)	53	Hemilaminectomy (tubular)	9.4 %	Clinical	14.8
	Chesney (2022) (Chesney et al., 2022)	85	Contralateral hemilaminectomy (tubular)	18.8 %	Clinical	46
	Kulkarni (2017) (Kulkarni et al., 2017)	30	Contralateral hemilaminectomy (tubular)	0 %	Clinical + X-ray	46.5
	Sukkarieh (2015) (Sukkarieh et al., 2015)	13	Contralateral hemilaminectomy (tubular)	0 %	Clinical	20.8
**Endoscopy**	Akbary (2020) (Akbary et al., 2020)	13	Endoscopic interlaminar contralateral	0 %	Clinical + MRI	12
	Hwang (2017) (Hwang et al., 2017)	14	Endoscopic interlaminar contralateral	0 %	Clinical + ODI	6
	Komp (2014) (Komp et al., 2014)	74	Endoscopic (foraminal/interlaminar)	2.7 %	Clinical	24
	Tacconi (2020) (Tacconi et al., 2020)	35	Endoscopic (foraminal/interlaminar)	5.7 %	Clinical + X-ray	15
	Oertel (2017) (Oertel and Burkhardt, 2017)	11	Endoscopic interlaminar ipsilateral	9.1 %	Clinical + ODI	10.5
	Vergara (2017) (Vergara et al., 2017)	13	Laminectomy	7.6 %	Clinical	14.9
	Epstein (2004) (Epstein, 2004)	35	Laminectomy	14.3 %	Clinical + SF-36	24

### 4.1 Surgical procedures and invasiveness

In the current day and age, several non-instrumented surgical procedures can be performed when treating synovial cysts with a varying degree of invasiveness. In simple terms, these can be divided into laminectomy (resection of bilateral vertebral laminae, spinous process, flavar and interspinous ligaments), hemilaminectomy open/tubular (resection of a unilateral vertebral lamina and ligamentum flavum) and endoscopic resection interlaminar or foraminal (a variable degree of lamina and facet drilling in order to place an endoscope with the spinal canal). Additionally, a variable amount of the facet joint resection is usually necessary to gain sufficient access to the cyst. Unfortunately, many studies tend to group several types of non-instrumented procedures into the category of ‘decompression’ albeit the invasiveness of each procedure is fundamentally variable in terms of preserving segmental stability. To make matters more complicated, the degree of local invasiveness varies even within individual decompression categories as individual surgeons have personally modified these techniques. In order to address this source of potential bias, the surgical procedure in our study was standardized in terms of using a unilateral microscopic approach and performing a complete or partial hemilaminectomy and partial facetectomy. Nonetheless, individual cases required a variable degree of facet joint resection based on cyst size and location. This was then quantified by comparing preoperative and postoperative MRI scans in order to gain an objective measurement of surgical invasiveness (Fig. 1). We used a value of 50 % facet joint resection as a cut-off to divide surgeries into more and less invasive procedures. This was based on biomechanical cadaveric research of Abumi et al., who demonstrated that unilateral medial facetectomy did not significantly affect segmental range of motion, whereas unilateral total facetectomy resulted in increased axial range of motion and biomechanical instability (Abumi et al., 1990). Furthermore, cysts were quantified based on their volume, as we expected larger cysts to require more invasive surgical approaches. Nonetheless the degree of facet joint resection and cyst volume did not significantly affect any of the dependent variables including new instability or listhesis.

### 4.2 Preoperative instability

In our view, determining the stability of a spinal motion segment containing the synovial cyst, is a key step in determining whether spine instrumentation is necessary. Unfortunately, there is currently no generally accepted definition of instability, which was recently shown in a surgeon survey (Spina et al., 2019). The lack of a clear definition results in heterogenous patient cohorts between studies, as some surgeons are prone to be more aggressive with spine instrumentation in borderline cases. We recognize that instability is made up two components – a dynamic component defined by postural changes in vertebral translation and/or angulation and a static component made consisting of disc height, degree of disc degeneration, facet joint effusions, presence of listhesis, patient age or even the presence of a synovial cyst. In our study, we only considered the presence of dynamic instability an indication for spine fusion. Components of static instability were used as independent variables as their role in cyst recurrence and/or development of instability in unclear. Our results demonstrated that the only independent variable of static instability affecting dependent variables was preoperative spondylolisthesis. Spondylolisthesis commonly accompanies synovial cysts, with Bruder et al. reporting a coincidence of 31 % in their review of 2900 cysts (Bruder et al., 2017). Several studies support the notion that spondylolisthesis is risk factor of future instability, listhesis progression and revision surgery in cases of synovial cyst resection (Campbell et al., 2018; Epstein, 2004). Campbell et al. specifically based their grading system on the degree of preoperative spondylolisthesis and spinal canal stenosis and demonstrated that higher grades are associated with cyst recurrence, revision surgery, worse ODI scores. These results suggest that spine instrumentation could be beneficial in these cases where a synovial cyst is present within a motion segment with spondylolisthesis. On the other hand, several studies had opposite findings with no correlation of preoperative listhesis with reoperation rate and cyst recurrence (Page et al., 2021; Xu et al., 2010; Scholz et al., 2017). Although preoperative spondylolisthesis did affect listhesis progression, new instability, adjacent segment stenosis and postoperative ODI, it did not ultimately not correlate with reoperation rate in our study. Overall, it was present in 36 % of patients who did not require an instrumented procedure. As such, we consider the presence of preoperative listhesis to be a warning sign indicating a higher probability of post-surgical instability and other static signs of instability should be analyzed to determine whether instrumentation is necessary.

### 4.3 Follow-up and postoperative imaging

The length of follow-up naturally influences the rate of reported long-term postoperative complications including reoperation rate, cyst recurrence, iatrogenic instability or spondylolisthesis. Of the 33 major studies shown in Table 4, only 7 had a mean follow up of over 5 years (Page et al., 2021; Rolemberg Dantas et al., 2020; Wun et al., 2019; Bruder et al., 2017; Weiner et al., 2007; Scholz et al., 2017; Birch et al., 2016). Although authors such as Xu found no association between cyst recurrence and the length of follow-up, the earliest cyst recurrence in our cohort occurred after 21 months and on average 71.3 months after primary surgery (Xu et al., 2010). This demonstrates that iatrogenic instability and/or cyst recurrence may take years to manifest and may be compounded by natural degenerative changes in segments with preexisting spondylolisthesis and disc degeneration. Interestingly, we did not have any cases of early cyst recurrence or listhesis decompensation occurring within the first year of surgery, which could be explained by the fact all our patients underwent standing radiographs, which were compared to prone MRI scans as well as dynamic flexion/extension X-rays to rule out latent instability. Another factor influencing the reported rate of long-term complications is how follow-up is performed. The majority available of studies performed follow-up by means of a clinical exam or telephone call and thus reported their results via Mcnab or Odom criteria. Only 5 studies utilized standardized questionnaires such as the Oswestry Disability Index (ODI) or SF-36 (Bruder et al., 2017; Scholz et al., 2017; Hwang et al., 2017; Oertel and Burkhardt, 2017; Epstein, 2004). Furthermore, imaging studies were usually only performed in cases of persisting/recurrent symptoms, with only 6 studies globally utilizing postoperative dynamic X-rays and 2 studies performing postoperative MRIs (Kulkarni et al., 2017; Jankowski et al., 2012; Landi et al., 2012; Domenicucci et al., 2017; Akbary et al., 2020; Tacconi et al., 2020). In our study, we reported postoperative outcomes via the ODI as it allows objectified comparison of results between studies and even between patients undergoing various types of spine procedures. Postoperative imaging was mainly performed to quantify surgical invasiveness but additionally allowed detection of latent pathologies such as instability, adjacent segment stenosis or adjacent segment synovial cysts, which would otherwise remain undetected. Knowledge of these conditions not only provides a more complex perspective on chronic postoperative changes occurring in proximity of the operative field but also served as feedback for patients, who were advised to undergo targeted physiotherapy.

### 4.4 Clinical and graphical outcomes

In our study, we considered any repeated surgery of a spine segment previously containing a synovial cyst a revision procedure. As such, we report a revision rate of 13.2 %. This result is slightly higher compared to similarly sized studies such as Page et al., Campbell et al., Bruder et al. or Scholz et al., who respectively report revision rates of 10.5 %, 10.8 %, 11.3 % and 12.2 %. The most likely explanation is the use of postoperative imaging, which may have swayed us towards more aggressive surgical treatment of borderline cases presenting dominantly with back pain and cyst recurrence and/or instability. Although some authors of large studies such as Xu, Komp or Metellus report lower revision rates of 3 %, 2.7 % and 2.6 % this is most likely due to significantly shorter follow-ups (Xu et al., 2010; Metellus et al., 2006; Komp et al., 2014). In terms of clinical outcomes, 63 patients reported a postoperative ODI ≤40. Ten of the 13 remaining patients underwent revision surgery, after which their ODI decreased by a mean average of 13.3. Of the 3 remaining patients, two had multiple musculoskeletal disorders including high grade hip arthrosis, sacroiliac joint dysfunction and multiple fractures of the thoracic spine. The remaining patient had no significant pathology on follow-up imaging apart from excessive scar tissue within the surgical corridor and was referred to an algesiologist. Interestingly, follow-up MRI exams identified a new adjacent-level synovial cyst in 6 cases and new adjacent-level stenosis in 9 cases. The mean length of follow-up imaging in these cases was 121(71–177) months, which indicates that these findings are most likely a form of adjacent segment disease due to spontaneous fusion of the operated segment after many years. Although 12 of these cases were clinically asymptomatic, these findings suggest that adjacent segment disease may be a common long-term complication many years after synovial cyst resection. In fact, three patients in our cohort underwent microsurgical decompression of symptomatic adjacent level stenosis and it is highly likely that this number to increase with longer follow-up.

### 4.5 Limitations

Our study contains several limitations which must be considered when interpreting its results. Firstly, it is designed as a retrospective observational study, which does not compare various surgical modalities and lacks a control group. Therefore, conclusions cannot be made about efficacy of microsurgical hemilaminectomy compared to other surgical strategies such as endoscopic or fusion procedures. Furthermore, it does not compare our patient cohort to a matched group of patients with segmental instability, which were primarily treated via instrumentation. Additionally, although the surgical procedure itself was standardized, it was performed by several operating surgeons which could result in variable degrees of local invasiveness. Secondly, our study analyzes patients at different follow-up time intervals. This could affect the reliability of volumetric analysis of facet resection as patients with longer follow-up intervals are more likely to have ossification of the bone defects caused by the approach. Furthermore, patients with shorter follow-up have a higher likelihood of revision surgery in the future as our mean revision interval was 71.3 months. Thirdly, preoperative ODI values were not obtained, which limits the quantification of improvement after surgery. Fourthly, patients did not undergo a standardized long-term physiotherapy regimen, which could act as a potential source of bias. Finally, a total of 30 patients complained of residual radicular pain. Although the pain intensity was clearly lower than preoperative values, it is possible that an instrumented fusion procedure could be more effective in eliminating radicular pain caused by micromotion trauma of the nerve root surrounded by scar tissue.

## 5 Conclusion

In conclusion, our study demonstrated that long term results several years after synovial cyst resection via microscopic hemilaminectomy remain acceptable, with a reoperation rate of approximately 13 %. As such, we consider microsurgical resection a feasible first choice in patients without signs of dynamic instability as the reoperation rate is comparable to recurrent disc herniation, where non-instrumented procedures are the mainstay of surgical treatment (Huang et al., 2016). Nonetheless, the presence of preoperative stable spondylolisthesis should be considered a significant risk factor for future instability, listhesis progression and higher postoperative ODI, as demonstrated by our multivariate regression analysis. In these cases, we suggest considering other patient-specific risk factors of instability, such as intervertebral disc height, facet joint effusions or BMI, when deciding between instrumented and non-instrumented procedures. No other variable appeared to significantly affect clinical a graphical postoperative outcomes including the degree of facet drilling. Even so, we would naturally advocate performing the least invasive surgical procedure which the operating surgeon is comfortable with. Furthermore, our study demonstrated that synovial cyst resection may be associated with adjacent segment degeneration due to accelerated degeneration of the operated spine segment, which should be considered if a patient's symptoms recur years after their primary procedure. Future directions of research include comparison of microsurgical procedures to even less invasive endoscopic techniques, which although popular, currently lack sufficient follow-up periods.

## Declaration of generative AI and AI assisted technologies in the writing process

The authors declare that no AI technology was utilized in preparing the article including research and translational engines.

## Funding

This research was financially supported by the internal grant ‘Analysis of long-term spinal stability, following synovial cyst resection’ of Krajská Zdravotní a.s. IGA-KZ IG9-217111042, of which three authors (Jan Lodin M.D, Jan Terč M.D. and Petr Vachata M.D., PhD) were a part of. It was furthermore supported by the internal grant IGA-KZ-2022-1-8 of which one author (Alena Sejkorová M.D., PhD) was a part of.

## Declaration of competing interest

The authors declare the following financial interests/personal relationships which may be considered as potential competing interestsJan Lodin reports financial support and article publishing charges were provided by Masaryk Hospital in Usti nad Labem. If there are other authors, they declare that they have no known competing financial interests or personal relationships that could have appeared to influence the work reported in this paper.
